# Dietary iron and calcium intakes during pregnancy are associated with lower risk of prematurity, stillbirth and neonatal mortality among women in Tanzania

**DOI:** 10.1017/S1368980016002809

**Published:** 2016-11-07

**Authors:** Dominic Mosha, Enju Liu, Ellen Hertzmark, Grace Chan, Christopher Sudfeld, Honorati Masanja, Wafaie Fawzi

**Affiliations:** 1 Rufiji Health and Demographic Surveillance System, Ifakara Health Institute, Rufiji, PO Box 78373, Dar es Salaam, Tanzania; 2 Department of Global Health and Population, Harvard T.H. Chan School of Public Health, Boston, MA, USA; 3 Department of Epidemiology, Harvard T.H. Chan School of Public Health, Boston, MA, USA; 4 Department of Nutrition, Harvard T.H. Chan School of Public Health, Boston, MA, USA

**Keywords:** Diet, Iron, Calcium, Pregnancy, Prematurity, Stillbirth, Neonatal mortality

## Abstract

**Objective:**

Prematurity, stillbirth and other adverse birth outcomes remain major concerns in resource-limited settings. Poor dietary intake of micronutrients during pregnancy has been associated with increased risk of adverse outcomes. We determined the relationships between dietary Fe and Ca intakes during pregnancy and risks of adverse birth outcomes among HIV-negative women.

**Design:**

Women’s diet was assessed through repeated 24 h diet recalls in pregnancy. Mean intakes of total Fe, Fe from animal sources and Ca during pregnancy were examined in relation to adverse birth outcomes and neonatal mortality. Women were prescribed daily Fe supplements as per standard perinatal care.

**Setting:**

Dar es Salaam, Tanzania.

**Subjects:**

A cohort of 7634 pregnant women.

**Results:**

Median (interquartile range) daily dietary intake of total Fe, animal Fe and Ca was 11·9 (9·3–14·7), 0·5 (0–1·1) and 383·9 (187·4–741·2) mg, respectively. Total Fe intake was significantly associated with reduced risk of stillbirth (trend over quartiles, *P*=0·010). Animal Fe intake was significantly associated with reduced risk of preterm birth and extreme preterm birth. Animal Fe intake was inversely related to neonatal mortality risk; compared with women in the lowest intake quartile, those in the top quartile were 0·51 times as likely to have neonatal death (95 % CI 0·33, 0·77). Higher Ca intake was associated with reduced risk of preterm birth (relative risk; 95 % CI: 0·76; 0·65, 0·88) and extreme preterm birth (0·63; 0·47, 0·86). Women in the highest Ca intake quartile had reduced risk of neonatal mortality (0·59; 0·37, 0·92).

**Conclusions:**

Daily dietary Fe and Ca intakes among pregnant women are very low. Improvement of women’s diet quality during gestation is likely to improve the risks of adverse birth outcomes.

Approximately two billion people in the world, mainly women and children, are estimated to have micronutrient deficiencies^(^ ^1^ ^)^. Maternal nutritional deficiencies are important contributors to adverse birth outcomes such as low birth weight (LBW), stillbirth, preterm birth and early infant death observed in developing countries^(^ ^2^ ^–^ ^4^ ^)^. Stillbirth and prematurity are important perinatal health problems worldwide, with the highest prevalence in sub-Saharan Africa and South Asia^(^ ^5^ ^)^. A baby born prematurely is at increased risk of both early and late complications, such as cerebral palsy, mental retardation, sensory deficits, respiratory and gastrointestinal problems^(^ ^6^ ^)^. No effective and affordable interventions are known to prevent prematurity in developing countries.

Fe deficiency, a primary cause of anaemia, is common in pregnancy and is associated with adverse pregnancy outcomes^(^ ^7^ ^)^. Requirements for dietary Fe are increased during pregnancy in order to support the expansion of red cell mass, the placenta and fetal demands^(^ ^8^ ^)^. In order to reduce the magnitude of Fe deficiency in pregnancy, the WHO recommends Fe and folic acid supplementations for pregnant women, especially in developing countries^(^ ^9^ ^)^. Evidence supports significant beneficial effects of Fe intake in early pregnancy, but this is logistically difficult as women in developing countries often start attending antenatal clinics late in pregnancy^(^ ^10^ ^,^ ^11^ ^)^. Dietary approaches to improve Fe status may be an additional effective and sustainable means to prevent and correct Fe deficiency; however, data on the relationships of dietary Fe intake with birth outcomes in developing countries are limited.

Mean risk of dietary Ca deficiency in Africa is 54 %, with the highest odds in pregnant women and children^(^ ^12^ ^)^. Ca deficiency in pregnancy is due to physiological changes; increased demand from the growing fetus and maternal urinary excretion of Ca are approximately twice as high during this period^(^ ^13^ ^)^. Some key important benefits of Ca in pregnancy include less gestational hypertension, LBW and preterm birth^(^ ^14^ ^)^. Routine Ca supplementation in pregnancy is recommended by the WHO, but currently few national plans include routine Ca supplementation as standard of care, partly due to cost and logistical issues^(^ ^15^ ^)^.

The present study examined the relationships of dietary Fe and Ca intakes during pregnancy with LBW, preterm birth, small for gestational age, stillbirth and early child mortality. We utilized data obtained from HIV-negative pregnant women who were enrolled in a multivitamin supplementation study in Tanzania.

## Methods

We conducted a prospective cohort study among pregnant women enrolled in a randomized, double-blind, placebo-controlled trial of daily prenatal multivitamin supplements. Pregnant women were recruited from nine public health centres in Dar es Salaam, Tanzania, from August 2001 to July 2004. Eligibility criteria included a negative test for HIV infection, willingness to attend the antenatal clinic monthly in study facilities until delivery and estimated gestational age between 12 and 27 weeks according to the last normal menstrual period. Randomized pregnant women were instructed to take a single daily dose of the supplement or placebo tablets from the time of enrolment in the second trimester until 6 weeks post-delivery. Multivitamin supplements included 20 mg thiamin, 20 mg riboflavin, 25 mg vitamin B_6_, 100 mg niacin, 50 µg vitamin B_12_, 500 mg vitamin C, 30 mg vitamin E and 0·8 mg folic acid. All women received daily doses of Fe (60 mg of elemental Fe), folic acid (0·25 mg) and sulfadoxine-pyrimethamine for malaria intermittent preventive treatment as standard of care. Further details about the study have been described elsewhere^(^ ^16^ ^)^.

A baseline questionnaire that included sociodemographic characteristics, medical and obstetrics history was completed. Dietary intake was assessed by 24 h recalls during monthly antenatal clinic visits from enrolment until 36 weeks of gestational age. Interviews were conducted by research nurses trained on foods and nutrition assessment. The multiple-pass recall approach was used; pregnant women were asked to remember and report freely all foods and beverages consumed in the preceding day. The latter was followed by guiding the respondents to recall eating occasions and times for each specific food consumed. This was accompanied by probing and detailed questions to determine the portion size of food intake, aided by a locally adopted food atlas. The *Tanzania Food Composition Tables* ^(^ ^17^ ^)^ were used to determine nutrient content and quantity.

Women were always encouraged to come to Muhimbili National Hospital for delivery, where they were attended by research midwives. Newborns were weighed immediately after birth.

The study protocol was approved by independent institutional review boards at Muhimbili University of Health and Allied Sciences and the National Institute for Medical Research in Tanzania and the Harvard School of Public Health in the USA.

### Study end points

The study’s end points included neonatal death (death within the first 28 d of life) and stillbirth (fetal death after 28 completed weeks of pregnancy). Other end points assessed were LBW (<2500 g), preterm delivery (birth before 37 weeks of gestational age), extremely preterm delivery (birth before 34 weeks of gestational age) and small for gestational age (birth weight below the 10th percentile for gestational age, according to INTERGROWTH)^(^ ^18^ ^)^.

### Statistical analysis

Data from the participants with at least one 24 h diet recall were included in the analysis. A mean prenatal dietary intake was estimated as the average of multiple 24 h dietary recalls. Fe sources from meat, fish and poultry were categorized under one group of dietary animal Fe. The mean total dietary Fe, animal Fe and Ca intakes were predictors of the study. The predictors were categorized into four groups based on quartiles: <25th percentile, 25·0–49·9th percentile, 50·0–74·9th percentile and ≥75th percentile.

Missing data of study variables were retained in the analysis using the missing-indicator method^(^ ^19^ ^)^. Log-binomial regression models were used to estimate the relative risk (RR) of each outcome for the higher levels of intake, compared with the lowest level of intake. Relationships between each potential confounder and end points were assessed by bivariate analysis. Predictors with a *P* value <0·2 in bivariate analysis were included in the multivariate analysis.

In subgroup analyses we tested for interactions between dietary intakes and multivitamin regimen on the outcomes of interest using the Mantel–Haenszel test. There were no statistical interactions between multivitamin regimen and dietary intakes on study outcomes; therefore we present the findings of the associations between dietary intakes and outcomes across both intervention arms. We evaluated the associations across quartiles of nutrient (total Fe, animal Fe and Ca) intakes with adverse birth outcomes and neonatal mortality using the non-parametric test for trend across order groups (nptrend).

All covariates were considered in the analysis. Continuous variables are presented as mean and standard deviation, and as median and interquartile range for skewed distribution. Categorical variables are summarized as percentages. Two-sided Wald test *P* values are presented. A Filmer–Pritchett wealth index based on family possessions was constructed^(^ ^20^ ^)^. The statistical software package Stata^®^ 12.0 was used for data analysis.

## Results

Of the 8468 pregnant women enrolled in the study, 7634 (90·1 %) women had known birth outcome and 24 h diet recall information, and hence were included in the current prospective cohort analysis. The median (interquartile range) number of times women were interviewed for 24 h recall was 3 (2–3). Within-subject CV for dietary Fe and Ca intake was 0·45 (95 % CI 0·45, 0·46) and 1·30 (95 % CI 1·27, 1·33), respectively. The median (interquartile range) daily intake of total dietary Fe, Fe from animal sources and Ca was 11·9 (9·3–14·7), 0·5 (0–1·1) and 383·9 (187·4–741·2) mg, respectively. Ninety-nine per cent (*n* 7602) of women had total daily dietary Fe intake below the RDA of 27 mg and 90·1 % (*n* 6880) of women had daily dietary Ca intake below 1200 mg. The mean (sd; range) gestational age at enrolment in the study was 21·2 (3·5; 8·4–27·0) weeks and for maternal age was 25·2 (5·1; 13·5–45·5) years. Baseline and demographic characteristics are presented in Table 1.

**Table 1:** Basic characteristics of the study participants: HIV-negative pregnant women recruited from nine public health centres in Dar es Salaam, Tanzania, from August 2001 to July 2004

Characteristic	*n*	%
Education (years)		
0–7	6183	78·0
8–11	1335	16·8
≥12	410	5·2
Age (years)		
<20	4219	55·6
25–35	3004	39·5
>35	369	4·9
Parity		
0 (primigravida)	3530	44·5
1	2224	28·1
2	1179	14·9
≥3	992	12·5
BMI category		
<22·0 kg/m^2^	1853	26·7
22·0–24·9 kg/m^2^	2417	34·8
25·0–29·9 kg/m^2^	2007	28·8
≥30·0 kg/m^2^	675	9·7
Hb (g/l)		
<85	2244	32·7
85–109	3788	55·2
≥110	829	12·1
	Median	Interquartile range
Daily total Fe intake (mg)	11·9	9·3–14·7
Daily animal Fe intake (mg)	0·5	0–1·1
Daily Ca intake (mg)	383·9	187·4–741·2
Energy intake (kJ/d)	9192	7550–10 899
Energy intake (kcal/d)	2196·9	1804·4–2605·0
Filmer–Pritchett wealth score	1·7	1·1–2·5

The mean (sd) birth weight among the cohort was 3143·5 (493·9) g, with 441 (6·2 %) of births being LBW (<2500 g). The mean (sd) gestational age was 39·6 (2·9) weeks with 1100 (14·9 %) preterm births (<37 weeks) and 298 (4 %) extreme preterm births (<34 weeks). There were 241 (3·1 %) stillbirths and 175 (2·4 %) neonatal deaths.

The relationships between total Fe intake and adverse birth outcomes and neonatal mortality are presented in Table 2. Total dietary Fe intake was not associated with prematurity or severe prematurity. Dietary Fe intake was associated with reduced risk of stillbirth in all upper quartiles (test for trend over quartiles, *P*=0·010). There was no significant association between total dietary Fe intake and the risk of LBW, small for gestational age or neonatal mortality.

**Table 2:** Total dietary iron intake in relation to adverse birth outcomes and neonatal mortality in HIV-negative pregnant women recruited from nine public health centres in Dar es Salaam, Tanzania, from August 2001 to July 2004

	Quartile of daily total Fe intake								
Outcome	1st (<9·30 mg)		2nd (9·30–11·94 mg)		3rd (11·95–14·70 mg)		4th (>14·70 mg)		*P* for trend
Low birth weight (<2500 g)									
No. at risk	1743		1780		1797		1790		
No. and % of cases	119	6·8	102	5·7	123	6·8	97	5·4	
Crude RR (95 % CI)	Ref.		0·84	0·65, 1·08	1·00	0·79, 1·28	0·79	0·61, 1·03	0·227
Adjusted RR (95 % CI)*	Ref.		0·84	0·65, 1·09	1·01	0·79, 1·28	0·78	0·60, 1·02	0·210
Preterm birth (<37 weeks)									
No. at risk	1816		1845		1850		1857		
No. and % of cases	296	16·3	244	13·2	257	13·9	303	16·3	
Crude RR (95 % CI)	Ref.		0·81	0·69, 0·95	0·85	0·73, 0·99	1·00	0·86, 1·16	0·827
Adjusted RR (95 % CI)*	Ref.		0·79	0·67, 0·92	0·80	0·69, 0·93	0·91	0·78, 1·05	0·297
Extreme preterm birth (<34 weeks)									
No. at risk	1816		1845		1850		1857		
No. and % of cases	77	4·2	79	4·3	64	3·5	78	4·2	
Crude RR (95 % CI)	Ref.		1·01	0·74, 1·37	0·81	0·59, 1·13	0·99	0·73, 1·35	0·649
Adjusted RR (95 % CI)*	Ref.		0·97	0·71, 1·31	0·76	0·55, 1·05	0·89	0·65, 1·21	0·244
Small for gestational age									
No. at risk	1705		1719		1746		1736		
No. and % of cases	196	11·5	181	10·5	188	10·8	198	11·4	
Crude RR (95 % CI)	Ref.		0·91	0·76, 1·11	0·94	0·77, 1·13	0·99	0·82, 1·19	0·996
Adjusted RR (95 % CI)*	Ref.		0·92	0·76, 1·12	0·94	0·78, 1·14	1·00	0·83, 1·21	0·913
Stillbirth									
No. at risk	1901		1900		1899		1909		
No. and % of cases	85	4·5	55	2·9	49	2·6	52	2·7	
Crude RR (95 % CI)	Ref.		0·65	0·46, 0·90	0·58	0·41, 0·81	0·61	0·43, 0·85	0·002
Adjusted RR (95 % CI)*	Ref.		0·67	0·48, 0·94	0·61	0·43, 0·87	0·66	0·47, 0·93	0·010
Neonatal death									
No. at risk	1743		1790		1789		1806		
No. and % of cases	46	2·6	43	2·4	49	2·7	37	2·1	
Crude RR (95 % CI)	Ref.		0·91	0·60, 1·37	1·04	0·70, 1·54	0·78	0·51, 1·19	0·380
Adjusted RR (95 % CI)*	Ref.		0·94	0·62, 1·41	1·10	0·74, 1·64	0·81	0·52, 1·25	0·525

RR, relative risk; ref., reference quartile.

* Adjusted for maternal education level, maternal age, BMI, parity, wealth, energy intake, baseline Hb level and treatment regimen.

Dietary animal Fe intake from meat, fish and poultry sources was assessed. The upper quartile means the one with highest intake (i.e. quartile 4) and the comparison or reference group is the lowest quartile. Dietary animal Fe intake was significantly associated with reduced risk of preterm birth: compared with the lowest quartile, women in the upper quartile were 0·75 times as likely to have preterm birth (95 % CI 0·65, 0·86; test for trend over quartiles, *P*<0·001). Similarly, comparing women in the highest and the lowest quartile of dietary animal Fe intake, the RR (95 % CI) of extreme preterm birth was 0·67 (0·51, 0·90; test for trend over quartiles, *P*=0·004). Intake of dietary animal Fe was inversely related to the risk of neonatal mortality; the adjusted RR (95 % CI) of neonatal mortality in the upper two quartiles was 0·62 (0·42, 0·91) and 0·51 (0·33, 0·77), respectively, while in the second lowest quartile it was 0·69 (0·46, 1·03). Dietary animal Fe intake was not significantly associated with reduced risk of LBW, small for gestational age or stillbirth (Table 3).

**Table 3:** Dietary animal iron intake in relation to adverse birth outcomes and neonatal mortality in HIV-negative pregnant women recruited from nine public health centres in Dar es Salaam, Tanzania, from August 2001 to July 2004

	Quartile of daily animal Fe intake								
Outcome	1st (0 mg)		2nd (>0–0·53 mg)		3rd (0·54–1·11 mg)		4th (>1·11 mg)		*P* for trend
Low birth weight (<2500 g)									
No. at risk	2160		1366		1784		1800		
No. and % of cases	152	7·0	66	4·8	119	6·7	104	5·8	
Crude RR (95 % CI)	Ref.		0·69	0·52, 0·91	0·95	0·75, 1·19	0·82	0·64, 1·04	0·293
Adjusted RR (95 % CI)*	Ref.		0·70	0·52, 0·92	0·96	0·76, 1·21	0·84	0·66, 1·07	0·384
Preterm birth (<37 weeks)									
No. at risk	2254		1408		1844		1862		
No. and % of cases	417	18·5	163	11·6	264	14·3	256	13·7	
Crude RR (95 % CI)	Ref.		0·62	0·53, 0·74	0·77	0·67, 0·89	0·74	0·64, 0·86	<0·001
Adjusted RR (95 % CI)*	Ref.		0·66	0·56, 0·78	0·80	0·69, 0·92	0·75	0·65, 0·86	<0·001
Extreme preterm birth (<34 weeks)									
No. at risk	2254		1408		1844		1862		
No. and % of cases	129	5·7	37	2·6	62	3·4	70	3·8	
Crude RR (95 % CI)	Ref.		0·46	0·32, 0·66	0·59	0·44, 0·79	0·66	0·49, 0·87	0·002
Adjusted RR (95 % CI)*	Ref.		0·49	0·34, 0·70	0·61	0·46, 0·83	0·67	0·51, 0·90	0·004
Small for gestational age									
No. at risk	2119		1315		1735		1737		
No. and % of cases	256	12·1	128	9·7	205	11·8	174	10·0	
Crude RR (95 % CI)	Ref.		0·80	0·66, 0·98	0·98	0·82, 1·16	0·83	0·69, 0·99	0·145
Adjusted RR (95 % CI)*	Ref.		0·82	0·67, 1·00	1·01	0·85, 1·20	0·87	0·72, 1·04	0·352
Stillbirth									
No. at risk	2333		1453		1905		1918		
No. and % of cases	79	3·4	45	3·1	61	3·2	56	2·9	
Crude RR (95 % CI)	Ref.		0·91	0·64, 1·31	0·94	0·68, 1·31	0·86	0·61, 1·21	0·435
Adjusted RR (95 % CI)*	Ref.		0·89	0·63, 1·29	0·92	0·66, 1·28	0·87	0·62, 1·22	0·859
Neonatal death									
No. at risk	2156		1380		1795		1797		
No. and % of cases	73	3·4	33	2·4	39	2·2	30	1·7	
Crude RR (95 % CI)	Ref.		0·71	0·47, 1·06	0·64	0·44, 0·94	0·49	0·32, 0·75	0·001
Adjusted RR (95 % CI)*	Ref.		0·69	0·46, 1·03	0·62	0·42, 0·91	0·51	0·33, 0·77	<0·006

RR, relative risk; ref., reference quartile.

* Adjusted for maternal education level, maternal age, BMI, parity, wealth, energy intake, baseline Hb level and treatment regimen.

The median (interquartile range) daily dietary Ca intake in the first, second, third and fourth quartile was 101·4 (67·0–144·30), 277·9 (230·7–329·0), 529·8 (4491·1–624·4) and 1078·3 (879·1–1426·8) mg, respectively. Dietary Ca intake was significantly associated with reduced risk of preterm birth in all upper quartiles (test for trend over quartiles, *P*<0·001). It was also significantly associated with reduced risk of extreme preterm birth by an average of 37–41 % (test for trend over quartiles, *P*=0·002). Dietary Ca intake was also significantly associated with reduced adjusted RR of LBW in the second and third quartiles. Intake of dietary Ca in the upper quartile was significantly associated with reduced risk of neonatal mortality (RR=0·59; 95 % CI 0·37, 0·92) compared with the lowest quartile. There was no significant association between dietary Ca intake and the risk of small for gestational age or stillbirth (Table 4).

**Table 4:** Dietary calcium intake in relation to adverse birth outcomes and neonatal mortality in HIV-negative pregnant women recruited from nine public health centres in Dar es Salaam, Tanzania, from August 2001 to July 2004

	Quartile of daily Ca intake								
Outcome	1st (<187·42 mg)		2nd (187·42–383·87 mg)		3rd (383·88–741·24 mg)		4th (>741·24 mg)		*P* for trend
Low birth weight (<2500 g)									
No. at risk	1765		1784		1774		1787		
No. and % of cases	132	7·5	101	5·7	93	5·2	115	6·4	
Crude RR (95 % CI)	Ref.		0·76	0·59, 0·97	0·70	0·54, 0·91	0·86	0·68, 1·09	0·169
Adjusted RR (95 % CI)*	Ref.		0·74	0·58, 0·95	0·69	0·54, 0·90	0·87	0·68¸ 1·10	0·204
Preterm birth (<37 weeks)									
No. at risk	1839		1850		1836		1843		
No. and % of cases	338	18·4	259	14·0	245	13·3	258	14·0	
Crude RR (95 % CI)	Ref.		0·76	0·66, 0·88	0·73	0·62, 0·84	0·76	0·66, 0·88	<0·001
Adjusted RR (95 % CI)*	Ref.		0·77	0·67, 0·89	0·74	0·63, 0·86	0·76	0·65, 0·88	<0·001
Extreme preterm birth (<34 weeks)									
No. at risk	1839		1850		1836		1843		
No. and % of cases	106	5·8	64	3·5	61	3·3	67	3·6	
Crude RR (95 % CI)	Ref.		0·60	0·44, 0·81	0·58	0·42, 0·78	0·63	0·47, 0·85	0·002
Adjusted RR (95 % CI)*	Ref.		0·62	0·46, 0·84	0·59	0·43, 0·80	0·63	0·47, 0·86	0·002
Small for gestational age									
No. at risk	1720		1737		1722		1727		
No. and % of cases	193	11·2	200	11·5	180	10·4	190	11·0	
Crude RR (95 % CI)	Ref.		1·02	0·85, 1·23	0·93	0·77, 1·13	0·98	0·81, 1·18	0·610
Adjusted RR (95 % CI)*	Ref.		1·00	0·83, 1·21	0·92	0·76, 1·12	0·99	0·82, 1·20	0·748
Stillbirth									
No. at risk	1904		1903		1901		1901		
No. and % of cases	65	3·4	53	2·8	65	3·4	58	3·0	
Crude RR (95 % CI)	Ref.		0·81	0·57, 1·16	1·00	0·71, 1·40	0·89	0·63, 1·27	0·800
Adjusted RR (95 % CI)	Ref.		0·82	0·57, 1·17	1·01	0·72, 1·42	0·92	0·65, 1·31	0·953
Neonatal death									
No. at risk	1766		1791		1785		1786		
No. and % of cases	49	2·8	50	2·8	47	2·6	29	1·6	
Crude RR (95 % CI)	Ref.		1·00	0·68, 1·48	0·95	0·64, 1·41	0·58	0·37¸ 0·92	0·027
Adjusted RR (95 % CI)*	Ref.		0·95	0·64, 1·39	0·91	0·61, 1·35	0·59	0·37, 0·92	0·029

RR, relative risk; ref., reference quartile.

* Adjusted for maternal education level, maternal age, BMI, parity, wealth, energy intake, baseline Hb level and treatment regimen.

## Discussion

Higher daily dietary intakes of Fe and Ca among Tanzanian women were significantly associated with reduced risks of preterm birth, extreme preterm birth and neonatal mortality. Dietary Fe intake also reduced the risk of stillbirth. These two micronutrients did not significantly reduce the risk of being small for gestational age.

Nearly all women included in the study had low daily dietary Fe intake, with a median of less than half the recommended amount in pregnancy (12 mg *v*. 27 mg). This is alarming considering that study participants resided in areas with high prevalence of malaria and helminthic infections, important causes of Fe deficiency in sub-Saharan African populations. Median daily dietary Ca intake was one-third when compared with the WHO recommended daily intake in pregnancy (384 mg *v*. 1200 mg)^(^ ^15^ ^,^ ^21^ ^)^. Despite the vast known advantages of Ca intake in pregnancy, it is not provided as a routine supplement in the prenatal health programme as recommended by the WHO^(^ ^15^ ^)^. The observed low intakes of Fe and Ca in the study may suggest the possibility of low intakes of other micronutrients that we did not assess. Similar reports of suboptimal intakes of most micronutrients in pregnancy have been presented in other studies^(^ ^22^ ^,^ ^23^ ^)^. Emphasis on interventions that promote intake of a diet rich in nutrients may have a significant impact on maternal nutrition because such a dietary approach is more sustainable and may benefit a woman even before conception. It is therefore not effective enough to underpin supplements uptake and ignore the promotion of a nutrient-rich diet considering that in many developing countries supplements are often provided in advanced pregnancy stages due to late booking at the antenatal clinic.

Mothers with higher dietary Fe intake from animal sources, as opposed to total dietary Fe intake, appeared to carry a significant risk reduction of preterm birth and extreme preterm birth. The direct link between maternal Fe intake and fetal maturity may be due to improved Hb level as a result of adequate Fe intake, which automatically improves fetal growth^(^ ^24^ ^)^. Recent studies support an association between maternal Fe supplementation and increased birth weight; improved birth weight is more of a linear dose response^(^ ^25^ ^,^ ^26^ ^)^. There are limited studies on the association between prenatal dietary Fe and birth outcomes. However, a study in Australia supports the association of a preconception diet rich in nutrients including Fe and reduced risk of preterm birth^(^ ^3^ ^)^.

Our study showed that higher total dietary Fe intake was protective against stillbirth. This may be due to the association between anaemia and low Fe intake, considering that anaemia in pregnancy has been reported to be a risk factor for stillbirth in a systematic review by Haider *et al*.^(^ ^25^ ^)^. To date, there are no effective interventions in place to prevent either stillbirth or preterm birth. Thus, it is vital to evaluate further the specific roles of Fe and other micronutrients in preventing stillbirth and preterm birth.

Fe intake in pregnancy has been associated with reduced risk of LBW^(^ ^25^ ^,^ ^27^ ^)^. A study in India showed that daily intake of Fe–folic acid supplements during pregnancy increases birth weight by 6·5 g per month^(^ ^28^ ^)^. Our study found no relationship between dietary Fe intake in pregnancy and reduced risk of LBW. This may be due to the fact that all women had been provided Fe supplements as per standard of care in Tanzania. Additionally we had very low median and limited variability of daily dietary Fe intake among study participants. The findings in our study are also supported by a study in the UK that showed no association between dietary Fe and birth weight^(^ ^10^ ^)^. Such inconsistency to support the role of Fe on birth weight may also be due to limitations of 24 h dietary recall assessments, in which failure to recall diet accurately and the chance of consuming a non-typical diet during the day prior to the interview are common.

Increased risk of neonatal mortality was directly associated with decreased intake of dietary animal Fe. It is not clear why the association was observed to be significant for Fe intake from animal sources alone and not also for total dietary Fe. The reason may be due to haem Fe, for which meat, poultry and fish are major sources, being more bioavailable as compared with non-haem Fe^(^ ^29^ ^)^. Such observation should open a way forward to explore the importance of Fe from animal sources and hence to encourage its intake among pregnant women in developing countries. To the best of our knowledge, the present study is the first to assess dietary Fe and the risk of early child survival. However, a study in Indonesia reported that Fe–folic acid supplementation in pregnancy reduces the risk of early neonatal deaths^(^ ^30^ ^)^. The biological relationship on this may be linked with the beneficial effects of Fe and folic acid use in pregnancy on risk reduction of preterm birth and LBW^(^ ^31^ ^)^. Preterm birth and LBW are reported to be important causes of neonatal death in developing countries^(^ ^32^ ^,^ ^33^ ^)^. Typically study participants are provided with Fe supplements in the antenatal clinic, although compliance with the supplements is variable given gastrointestinal adverse effects^(^ ^34^ ^)^. The specific benefits of higher dietary Fe intake were noted in spite of women receiving prenatal supplements at the initiation of antenatal clinic visits. Therefore, the beneficial role of dietary Fe intake on pregnancy outcome should not be underestimated.

Ca is known as one of the vital micronutrients for the pregnant woman and her growing fetus. Yet, the role of Ca intake in pregnancy in relation to birth outcomes is not clear, with conflicting results on its advantages and risk reduction of LBW and preterm birth^(^ ^14^ ^)^. Our present study showed dietary Ca intake was significantly associated with reduced risk of both preterm birth and extreme preterm birth. This is also supported by Ramakrishnan *et al*. in their meta-analysis review of Ca supplementation^(^ ^35^ ^)^. The role of prenatal Ca intake in relation to reduced risk of preterm birth may be associated with its function in supporting fetal growth and maturity. This may also explain the significant association observed in our study between higher dietary Ca intake and reduced risk of neonatal mortality.

Despite low median daily dietary Ca intake among study participants, dietary Ca intake was borderline significantly associated with reduced risk of LBW. This may agree with a study in Iran that showed the association between maternal Ca intake and increased birth weight^(^ ^36^ ^)^. The association is particularly clear when Ca intake reaches dietary recommendation levels^(^ ^37^ ^)^, but the latter was not the case in our study. The advantage of Ca intake to the human body also depends on optimal levels of vitamin D. Our study’s ability to determine multiple interaction effects among different micronutrients and vitamins in relation to birth outcomes and early child survival was limited. Since the mean amounts of daily dietary Fe and Ca intakes were very low in our study, their role in reducing risks of LBW, preterm birth, small for gestational and neonatal mortality may have been significantly higher than what we observed if it was in a population with adequate intake of these micronutrients. Furthermore, limitations of dietary intake studies, particularly imprecise estimates of nutrient intakes and fewer numbers of times to interview women for 24 h recall, should not be ignored when evaluating nutritional net benefits. However, these limitations contribute to random misclassification of dietary nutrient intakes, which tends to bias results towards the null rather than present associations that are not real.

## Conclusion

Daily dietary Fe and Ca intakes among Tanzanian pregnant women in Dar es Salaam were very low compared with the WHO recommended levels. We determined that dietary Fe and Ca were associated with reduced risk of low preterm birth, stillbirth and neonatal mortality. Dietary micronutrients in pregnancy may be vital, particularly in developing countries where indicators of maternal and child health are worse. Improvement of women’s diet quality during gestation is likely to improve the risks of adverse birth outcomes. Further research on dietary intervention is needed to assess the effects of individual micronutrients, preconception and during gestation, in relation to pregnancy and early child outcomes.
